# An overview of key industrial product citric acid production by *Aspergillus niger* and its application

**DOI:** 10.1093/jimb/kuaf007

**Published:** 2025-03-28

**Authors:** Ambreen Latif, Noor Hassan, Hazrat Ali, Muhammad Bilal Khan Niazi, Zaib Jahan, Iqra Latif Ghuman, Farwa Hassan, Anam Saqib

**Affiliations:** Industrial Biotechnology Division, National Institute for Biotechnology and Genetics Engineering-College (NIBGE-C), Pakistan Institute of Engineering & Applied Sciences (PIEAS), Faisalabad, Pakistan; School of Chemical & Materials Engineering, National University for Science and Technology, Islamabad, Pakistan; Industrial Biotechnology Division, National Institute for Biotechnology and Genetics Engineering-College (NIBGE-C), Pakistan Institute of Engineering & Applied Sciences (PIEAS), Faisalabad, Pakistan; Industrial Biotechnology Division, National Institute for Biotechnology and Genetics Engineering-College (NIBGE-C), Pakistan Institute of Engineering & Applied Sciences (PIEAS), Faisalabad, Pakistan; School of Chemical & Materials Engineering, National University for Science and Technology, Islamabad, Pakistan; Department of Chemical Engineering, King Fahd University of Petroleum and Minerals (KFUPM), Dhahran, Saudi Arabia; Interdisciplinary Research Center for Refining & Advanced Chemicals, King Fahd University of Petroleum and Minerals (KFUPM), Dhahran, Saudi Arabia; School of Chemical & Materials Engineering, National University for Science and Technology, Islamabad, Pakistan; Industrial Biotechnology Division, National Institute for Biotechnology and Genetics Engineering-College (NIBGE-C), Pakistan Institute of Engineering & Applied Sciences (PIEAS), Faisalabad, Pakistan; Industrial Biotechnology Division, National Institute for Biotechnology and Genetics Engineering-College (NIBGE-C), Pakistan Institute of Engineering & Applied Sciences (PIEAS), Faisalabad, Pakistan; QuinTech Center for Applied Sciences, Lahore, Pakistan

**Keywords:** *A. niger*, Citric acid, Molecular biology, Industry, Fermentation

## Abstract

Citric acid possesses high economic value and is considered as the world’s largest consumed organic acid in numerous industries. Citric acid applications range from food to beverage industries, pharmaceuticals, cosmetics, and the environment. It is mostly produced by microbial fermentation, but *Aspergillus niger* is considered as the main workhorse for large-scale production of citric acid. In the current review, special devotion has been made toward addressing the latest and innovative literature related to production of citric acid by *A. niger*. The review article discusses *A. niger* historical involvement in citric acid production, fermentation technologies, molecular biology, biosynthesis, accumulation of citric acid, methods for enhanced production of citric acid, different operational factors also influencing citric acid production, and various techniques used for citric acid recovery. Also, copious biotechnological applications of citric acid are summarized for a fundamental comprehension of the subject and its critical role in diverse fields of industries.

**One-Sentence Summary:**

This review describes the historical role of *Aspergillus niger* in the production of citric acid, fermentation technologies, molecular biology, techniques for increased citric acid production, and other physical and chemical variables influencing the production of citric acid.

## Graphical Abstract

**Graphical Abstract:**
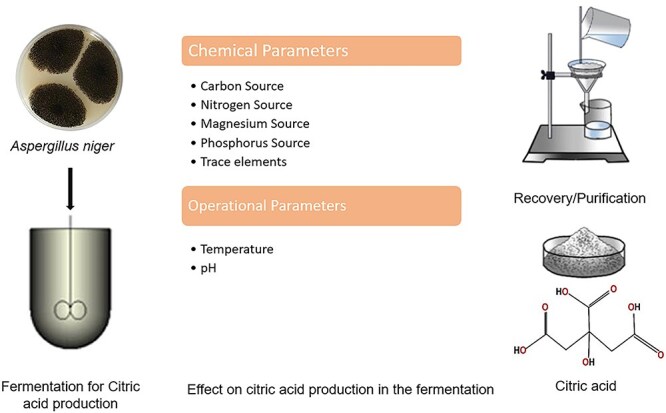

## Introduction

**Fig. 1.**
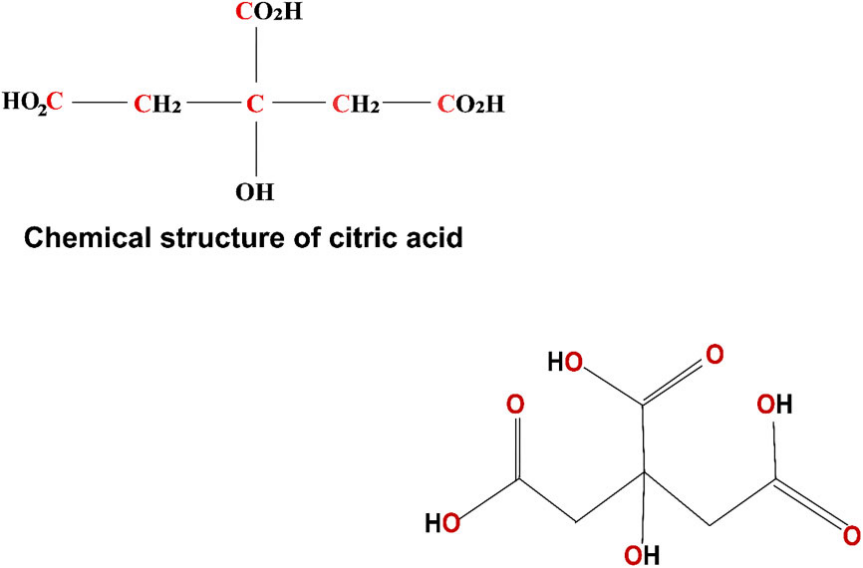
Chemical structure of citric acid, characterized by three –COOH groups.

The word “citric” or 2-hydroxy-propane-1,2,3-tricarboxylic acid is derived from the Latin term *citrus*, which denotes trees of the genus *Citrus*, including lemon trees (Max et al., 2010). Citric acid is a weak organic acid that is colorless and easily water-soluble in pure form. It is also known as tribasic based on the presence of tricarboxylic acid functional groups (Fig. 1), thus providing three pKa values, which are 3.13, 4.76, and 6.39 (Sinko, 2023). Citric acid exhibits a solid phase at room temperature, with boiling and melting points of 310°C and 153°C, respectively (Kubicek, 1998). It exists either as anhydrous or in monohydrate form, providing molecular weights of 192.12 and 210.14, respectively. Both forms of citric acid are soluble in water. However, anhydrous citric acid is also freely soluble in ethanol, whereas monohydrate citric acid is frugally ether soluble.

Different natural or synthetic sources are used for citric acid production. Globally, citric acid production has risen to 2.39 million tons in 2020, which is estimated to increase to 2.91 million tons in 2026 (Lambros et al., 2022). Citric acid has a large number of applications in different industries (e.g. food, pharmaceutical, and cosmetics). As an acidulant, citric acid is applied in food sector due to high solubility and lower toxicity and is also used in cleaning of special boilers. Furthermore, a bulk amount of citric acid is used in beverages, food, and confectionery industry as an antioxidant, emulsifier, buffer, chelator, flavor enhancer, and acidulant (Behera, 2020). In some scenarios, citric acid is replacing phosphate to enhance power of detergents. Due to this reason, it is not only used for cleaning metals in industries but also used in homemade detergents. Citric acid can produce chelate complexes with metal ions. So, citric acid assists in stabilizing oil and fat due to oxidation reactions of ion catalyzed (Lambros et al., 2022).

Citric acid can be produced by either extracting from various natural sources such as lime, lemon, and orange or microbial fermentation. However, the majority of plants are unable to generate enough quantities that are demanded in the market. Therefore, many microorganisms are currently applied for large-scale citric acid production. These microbes include members from both bacteria and fungi (e.g., *Bacillus licheniformis, Arthrobacter paraffinens, Penicillium janthinellum, Candida* species, *Yarrowia lipolytica, Hansenula anamola*, and species of genus *Aspergillus*) (Mores et al. 2021). Selection of microbial strains/species could significantly influence the fermentation process. For example, Pazouki and Panda (1998) stated a citric acid production of 0.09 g/g from cane sugar molasses using *Candida parapsilosis* NH-3. However, *Aspergillus niger* has more popularity and advantages among all these microorganisms for bulk production of citric acid because of its ability to use a wide range of raw materials and high production of citric acid (Soccol et al., 2006). *Aspergillus niger* demonstrates a capacity to produce a maximum of 90 g/l of citric acid through submerged fermentation when applying sucrose as a substrate (Chmiel, 1975).

In the last 70 years, much more research has been done to increase citric acid production because citric acid is extensively applied in copious industries such as food and beverages, pharmaceuticals, cosmetics, and textiles. In this review, citric acid production from *A. niger* is discussed in detail. In addition, several fermentation techniques and associated parameters influencing citric acid production, and various extraction protocols for citric acid have also been reviewed. This review also describes metabolic engineering used for enhancing the production of citric acid from *A. niger*.

### *Aspergillus*  *niger*: The Suitable and Best Selection for Citric Acid Production

Suitability and selection of microbes for citric acid production are based on having a higher tendency for citrate synthase activity but lower activity for isocitrate dehydrogenase and aconitase hydratase as citrate synthase initiates citric acid cycle or Krebs cycle or tricarboxylic acid (TCA) cycle. The highest activity of citrate synthase suggests that microbes are more proficient at releasing citrate, a crucial intermediate in the cycle. Citrate serves as a precursor for energy production via oxidative phosphorylation. The citrate synthase activates the condensation initiation of acetyl-CoA and oxaloacetate to release citrate, which is the first and utmost step in the Krebs cycle. Conversely, isocitrate dehydrogenase transforms isocitrate into α-ketoglutarate, while aconitase hydratase converts citrate to isocitrate. Diminished enzyme activity in these cases could indicate that the microbe is redirecting its metabolic flow from the Krebs cycle, potentially toward alternate pathways or metabolites (Książek, 2023; Prescott & Dunn, 1959). Among the potential microbes, the black species of *Aspergillus* (e.g. *A. niger*) have this property because it produces black-colored molds known as citric acid-producing cell factories (Khurshid et al., 2001; Pazouki et al., 2000; Show et al., 2015; Singh Dhillon et al., 2011; Yokoya, 1992). *Aspergillus niger* is more popular among all other microorganisms for bulk production of citric acid because of possessing the ability to use a wide range of raw materials (Lodhi et al., 2001). Wucherpfennig et al. (2011) reported that different primary and secondary metabolites (e.g.. proteins, enzymes, and organic acids) are produced by a filamentous fungus *A. niger* possessing great research and economic values.

*Aspergillus niger* has a large number of advantages as compared to other microorganisms such as high capacity of citric acid accumulation, easy handling, high yield of citric acid, capability to process various substrates for fermentation, and low fermentation cost (Pazouki et al., 2000; Yokoya, 1992). However, a strain of *A. niger* can be further improved by mutagenesis either by physical or chemical methods (Lotfy et al., 2007b; Rodrigues et al., 2009). The strain of *A. niger* can be stored at 4°C and grow efficiently between pH 2.5 and 3.5 (Ozdal & Kurbanoglu, 2019).

## Historical Aspects of Citric Acid Production by *A. niger*

In 1784, a well-known Swedish chemist, Karl Wilhelm Scheele, invented the method for extraction of citric acid from lime juice. Later, the same procedure was used in England around 1826 for citric acid production from lemon (Oiza et al., 2022). In 1893, Wehmer German, a botanist, started working on a sugar fermentation medium for production of citric acid using *Penicillium glaucum* (Kubicek 1998). After 2 years, he recovered two strains that could produce citric acid and named these strains *Citromyces* spp (*Penicillium*). However, these strains did not get good value for citric acid production at the industrial scale due to long fermentation time and contamination issues (Oiza et al., 2022).

In 1917, James Currie successfully used *A. niger* for economic commercial production of citric acid, which could grow in an acidic environment. Currie observed the maximum yield of citric acid within 9–12 days of fermentation by providing optimum conditions. Later, Pfizer took the initiative to produce citric acid commercially using surface fermentation technology (Sawant et al., 2018). In surface fermentation, the microorganism is grown on the surface of liquid. The first methodology for fermentation developed for citric acid production was surface fermentation, which still contributes to 20% of the world’s citric acid production (Papagianni, 2017). Agnihotri has predicted that *A. niger* can produce citric acid using 3-, 4-, 5-, 6-, and 12-carbon sugars, while the highest yield of citric acid is achieved using sucrose as a carbon source (Agnihotri, 1966). The metabolic product, citric acid, is produced during an intermediate stage of the Krebs cycle (Kubicek, 1998). It can be produced by fermentation technology in large amounts as it has a large number of applications in numerous industries. The increasing population has increased the consumption rate of citric acid. Time is needed to explore different substrates and methods to increase the quantity of citric acid.

### Fermentation Technologies for Citric Acid Production

Citric acid is obtained by a fermentation process. The fermentation process transforms sugar molecules into a new compound/product biologically in the presence of microorganisms. The most cost-effective and well-developed process for citric acid production is fermentation. Globally, more than 90% of the citric acid production is achieved by fermentation. This approach has several benefits such as less energy consumption, requiring a less complex control system and technical expertise, operating smoothly and steadily, and less chances of power plant failure (Soccol et al., 2006). Generally, preparation and its inoculation, specific technique of fermentation, and recovery of citric acid are three main steps included in the fermentation process.

**Fig. 2.**
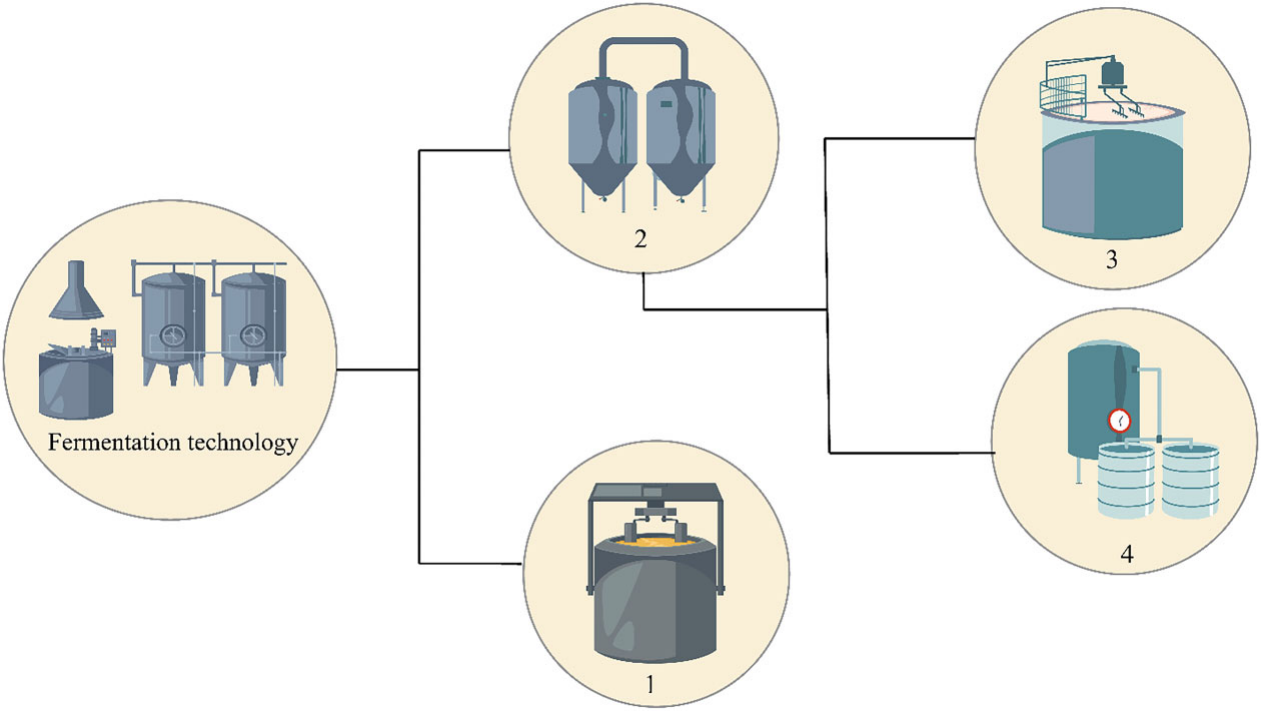
Classification of fermentation techniques: (1) solid substrate fermentation, (2) liquid substrate fermentation, (3) surface fermentation, and (4) submerged fermentation.

Two main types of fermentation processes are usually used for citric acid production, liquid- and solid-state processes. Liquid substrate fermentation is further divided into submerged and surface fermentation, as elaborated in Fig. 2. Each fermentation technique has different operational parameters, labor cost, and type of raw material, as stated in Table 1 (Behera, 2020; Max et al. 2010; Show et al., 2015).

**Table 1.** A Comparison of Different Fermentation Technologies

	Solid State Fermentation	Submerged Fermentation	Surface Fermentation
Contamination	Less risk	Less risk	High risk
Waste	Produce low waste	Less waste generation	**–**
Cost	Less operating cost	Less maintenance cost	Less energy cost
Operational parameters	Difficult to scale up a process, control process parameters, and poor heat transfer	Demand a more sophisticated installation, greater energy cost-rigorous control Most important is formation of foam	Labor-intensive, sensitive to changes in composition of the media
Raw material	Agro-industrial waste	Glucose syrup or beet molasses	Glucose syrup or beet molasses
Labor cost	Less labor cost	Less labor cost	High labor and maintenance costs
Citric acid production	High production but high impurity increases recovery cost	High yield	Low yield of citric acid

### Liquid Fermentation Technique

Liquid substrate fermentation involves a biotechnological method where microorganisms such as bacteria, yeast, or fungi are nurtured and raised within a liquid environment. The fluid medium, commonly known as substrate or broth, contains crucial nutrients required for the microorganisms’ growth and metabolism. Throughout the fermentation procedure, microorganisms utilize nutrients existing in a liquid substrate to perform diverse biochemical reactions resulting in the production of desired and highly specific products (Behera, 2020; Max et al., 2010). The technique of liquid substrate fermentation finds extensive application in producing a wide range of products, including enzymes, antibiotics, organic acids, biofuels, and other bioproducts. This process offers several advantages such as higher biomass yields, easier scalability, and the ability to control environmental conditions more precisely compared to solid-state fermentation (Behera, 2020).

### Submerged Fermentation Technique

Submerged fermentation is usually carried out in a liquid medium and microorganisms are suspended within a liquid medium. Precise control is required and widely used for large-scale production. Approximately 80% of the citric acid produced worldwide is attained by submerged fermentation (Kubicek, 1998; Mattey & Kristiansen, 1999; Pallares, 1996; Vandenberghe et al., 1999). *Aspergillus niger* demonstrates capacity to produce a maximum of 90 g/l of citric acid through submerged fermentation when utilizing sucrose as a substrate (Chmiel, 1975). Aeration and construction material of submerged fermenters are critical parameters for citric acid production. Low-capacity fermenters (<1 m^3^) and corrosion play actively as an outcome of increased surface-to-volume ratio. In this scenario, protective plastic players are used on steel-constructed fermenters. As compared to surface fermenters, submerged fermenters are less sensitive to varying media compositions like molasses, which have always different compositions. Glucose syrup or beet molasses substrates are widely used in submerged fermentation (Max et al., 2010; Papagianni, 2017).

Moreover, this technique has numerous advantages as compared to other techniques (e.g., less contamination risk, less labor cost, and a high yield of citric acid production) (Habison et al., 1983; Soccol & Vandenberghe, 2003; Soccol et al., 1999, 2006). Different types of substrates can be used in submerged fermentation as it provides good control of process conditions (Soccol et al., 2006). Fermentation of carbon-containing raw material is carried by anaerobic microorganisms under anoxic conditions (Lingappa et al., 2002; Swain et al., 2011). A pH below 2.0 in citric acid-producing media is the main advantage of low contamination risk. However, foam formation during submerged fermentation is the main problem that can be resolved by applying antifoaming agents such as vegetable or animal fats and mechanical systems (Max et al., 2010; Papagianni, 2017).

### Surface Fermentation

In surface fermentation, microorganisms are cultured on the surface of medium. The first fermentation technique developed for citric acid production was surface fermentation, which still contributes to 20% of world citric acid production (Papagianni, 2017). This fermentation technique is considered by a low yield of citric acid, no foam formation, and less energy consumption (Moyer, 1953). Surface fermenters consist of several trays and these trays are arranged into shelves. Culture media are placed on these trays, made of special-grade steel or polyethylene and high-purity aluminum. Surface fermentation requires high labor and maintenance costs because large laborers are required to clean trays and pipes (Rubio & Maldonado, 1995; Soccol & Vandenberghe, 2003; Soccol et al. 1999, 2006). Surface fermentation is also sensitive to varying compositions of production media (Benghazi et al., 2014). Proper aeration is needed for distributing air to microbes and controlling media temperature (Kubicek et al., 2016; Soccol et al., 2006).

### Solid-State Fermentation

Solid-state fermentation (SSF) is described as the biological transformation of carbon substrate into valuable products where the microorganism works on solid phase in the absence of mobile liquid phase (Pandey, 2003). When the final product is produced then it could be recovered or used as it is (Ballardo et al., 2020). Different reactors (e.g., performance of a tray, plug flow mechanically stirred reactor, and packed bed reactors) are useful in SSF (Arora et al., 2018; Carboué et al., 2019). Solid-state fermentation is also called koji process as it was first used for *Aspergillus oryzae* (also known as Super Koji). The first time, solid-state fermentation was started in Japan (Papagianni, 2007)*. Aspergillus niger* is grown on some insoluble materials that function as a good source of nutrients and support for microorganisms in solid-state fermentation (González-Sáiz et al., 1997; Pandey, 1992). The optimum temperature and pH are 28–30°C and 4.5–6.0, respectively. The required fermentation time for the solid-state fermentation technique is 4–5 days (Drysdale & McKay, 1995). In comparison to submerged fermentation, solid-state fermentation has various advantages compared to submerged fermentation (e.g., comfortable usage), utilizing economical and in-bulk available agro-industrial waste without passing through any tough pretreatment process (Berovic & Legisa, 2007). This technique has less risk of contamination, less energy consumption, produces less waste, and easy steps required for the recovery of citric acid. Anyhow, it has several drawbacks such as difficulty in process scale-up, poor heat transfers within media, and difficulty in controlling process parameters due to a solid phase (Kapilan, 2015). In solid-state fermentation, a large quantity of citric acid is produced using *A. niger*. Microorganisms that need low phosphorus and nitrogen are not used in solid-state fermentation (Hossain et al., 1984).

## Molecular Biology of Citric Acid Production by *A. niger*

The Krebs cycle is primarily regulated by nine genes: aconitase 2, citrate synthase, fumarate hydratase, malate dehydrogenase 1, oxoglutarate (α-ketoglutarate) dehydrogenase, pyruvate dehydrogenase (lipoamide) α-1, pyruvate dehydrogenase (lipoamide) α-2, succinate dehydrogenase complex, and succinate-CoA ligase.

A protein-coded gene named aconitase dehydrogenase has eight exons. A protein-coded gene named citrate synthase dehydrogenase has seven exons. A protein-coded gene named fumarate hydratase has six exons. A protein-coded gene named oxoglutarate dehydrogenase has five exons. A protein-coded gene named pyruvate dehydrogenase has four exons. A protein-coded gene named succinate dehydrogenase has six exons. A protein-coded gene named succinate CoA has eight exons. All of the above-mentioned genes are located on “chromosomes” (Nelson & Cox, 2017). The following transporters are involved in the Krebs cycle: pyruvate transporter (mitochondrial pyruvate carrier), citrate transporter (citrate carrier), malate-aspartate shuttle, α-ketoglutarate transporter (oxoglutarate carrier), dicarboxylate transporter, and adenine nucleotide translocase. All these genes and transporters collectively perform their role in the Krebs cycle (Palmieri, 2013).

During the glycolytic process, the generated pyruvate undergoes oxidation and associates with coenzyme A, resulting in the release of acetyl coenzyme A (acetyl-CoA), carbon dioxide (CO_2_), and nicotinamide adenine dinucleotide (NAD) plus hydrogen (H) in the form of NADH. Following this step, the acetyl-CoA combines with oxaloacetate, leading to the production of citrate. Furthermore, pyruvate, a byproduct of glycolysis, has the potential to undergo carboxylation mediated by pyruvate carboxylase, resulting in the formation of oxaloacetate. As acetyl-CoA combines with oxaloacetate, the resulting citrate undergoes a series of reactions that regenerate four-carbon oxaloacetate and release two carbon dioxide molecules. Subsequently, one molecule of acetic acid is released, and two adenosine triphosphate and CO_2_ molecules are produced. Later on, one oxaloacetate molecule is utilized to produce citrate (Książek., 2023; Xu et al., 2020; Xue et al., 2021).

## Biosynthesis of Citric Acid

The synthesis process of citric acid starts with a pyruvate molecule, which comes from sugar glycolysis. The glycolysis process in mitochondria produces two pyruvate molecules from one sugar molecule, which transports through membrane into matrix where the Krebs cycle starts. There it reacts with coenzyme A to release acetyl-CoA, NADH, and CO_2_. Acetyl-CoA further reacts with oxaloacetate and releases citrate. Besides, pyruvate molecules can be carboxylated to produce oxaloacetate for restocking the quantity in the Krebs cycle, so that increased demand of energy of cells can be fulfilled (Behera, 2021; Ratledge, 2001).

**Fig. 3.**
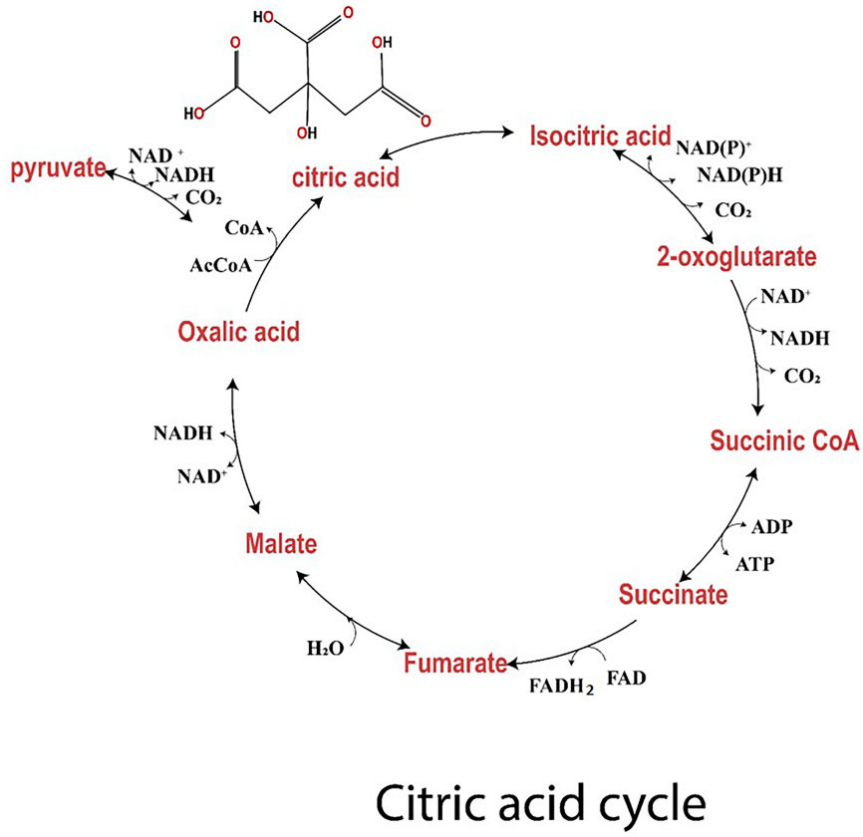
An overview of the main metabolic steps involved in citric acid production by *A. niger*.

Citric acid is produced as a result of a combination of oxaloacetate and acetyl-CoA, which passes through a chain of reactions producing two CO_2_ molecules and four carbon oxaloacetate molecules at the end of reaction, as shown in Fig. 3. During the second step, acetyl-CoA combines with oxaloacetate. Each cycle starts with one molecule of acetyl-CoA, producing two ATP molecules and CO_2_. One acetyl-CoA participates in citric acid synthesis (Behera, 2020). In mitochondria and cytoplasm of *A. niger*, NADP^+^-dependent enzyme isocitrate dehydrogenase is present, which is activated by Mg^2+^ or Mn^2+^ and inhibited by α-ketoglutarate and citrate resulting in citric acid accumulation (Ratledge, 2001).

## Accumulation of Citric Acid

**Fig. 4.**
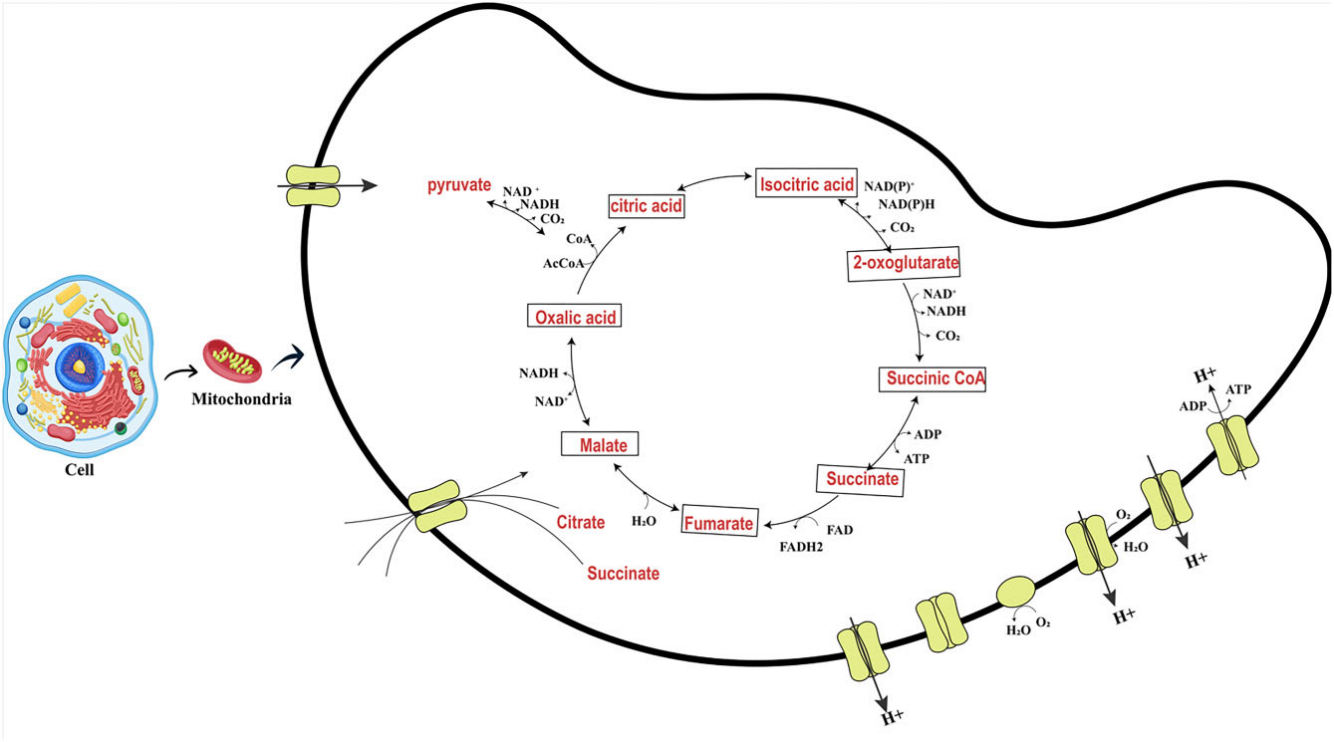
Production of citric acid by *A. nige r* inside mitochondria.

The regulation of *A. niger* metabolic pathways is highly effective in citric acid accumulation. Citric acid synthesis carries specific metabolic processes involved in citric acid synthesis that are interconnected with other cellular processes, and accumulation of citric acid can serve as a way to balance and regulate these metabolic pathways, which are interconnected with other cellular processes (Behera, 2020). It has been recommended that citric acid accumulation can be enhanced by deactivating enzyme activity like isocitrate dehydrogenase and aconitase that degrades citric acid in the Krebs cycle. In numerous studies, it has been investigated that the Krebs cycle is very active in producing its intermediate during citric acid production (Ahmed et al., 1972; Jernejc et al., 1992). As citric acid is produced inside fungal cells, it needs to be transported across the cell membrane for releasing to external environment. This transportation is facilitated by transport proteins embedded in cell membrane. For this purpose, dicarboxylate transporters are added to compete with aconitase enzyme to release citric acid from mitochondria instead of deactivating activity of the enzyme, as presented in Fig. 4.

Citric acid accumulation is affected by various enzymes and secretion in *A. niger*. The combination of acetyl-CoA with oxaloacetate produces citrate in the action of citrate synthase enzyme catalyzed by an initial step of the Krebs cycle. In *A. niger*, this enzyme is highly demanded for production of citric acid. Secondly, citrate is converted into isocitrate with the action of aconitase enzyme in the Krebs cycle. Aconitase is crucial for continuation of the Krebs cycle and producing isocitrate, a precursor for further steps in citric acid (CA) production. In the glyoxylate cycle, which is a variation of the Krebs cycle, the following enzymes isocitrate lyase and malate enable isocitrate to convert into glyoxylate and succinate. The glyoxylate cycle enables the fungus to bypass certain steps of the Krebs cycle and efficiently convert acetyl-CoA into succinate. This is an important step for CA production (Li et al., 2016; Muszkieta et al., 2014).

The mitochondria and cytoplasm of *A. niger* comprise NADP^+^-dependent enzyme isocitrate dehydrogenase, which is activated by Mg^2+^ or Mn^2+^ and inhibited by α-ketoglutarate and citrate resulting in citric acid accumulation (Ratledge, 2001). Furthermore, α-ketoglutarate is converted into succinyl-CoA in the presence of a complex set of enzymes in the Krebs cycle. This step generates high-energy molecules in form of NADH and flavin adenine dinucleotide (FADH_2_), which are essential for energy production and subsequent reactions in the cycle. Finally, not an enzyme, the citrate exporter protein is responsible for transporting citrate out of the fungal cell and into the surrounding medium. This step is critical for secretion of citric acid. The exporter helps to maintain a favorable concentration gradient by continuously removing citrate from cells, allowing for more citrate synthesis (Ratledge, 2001).

## Strategies for Enhancing Citric Acid Production

There are various methods to improve the strain tendency of *A. niger* for enhanced citric acid yield and production (e.g., chemical and physical mutagenesis and genetic engineering). Due to large demand for citric acid, strain improvement for higher citric acid production is mandatory considering following factors: the overall effectiveness of the fermentation process can be improved by optimizing yield and reducing production cost, high citric acid production rate, better substrate consumption, high resistance to inhibitory factors, and minimize by product formation.

However, mutation by a traditional approach like physical and chemical methods is difficult to establish as *A. niger* is a filamentous fungus and shows a complex association between production phase and morphology of microorganisms in submerged fermentation (Krull et al., 2010, 2013). However, all strains of *A. niger* do not possess capability to produce citric acid. Those *A. niger* strains that exhibit citric acid production capabilities have undergone genetic modifications, enabling them to secrete elevated amounts of citric acid (Xu et al., 2020). Keeping in mind, above-mentioned mutation is risky because this mutation can lead to either a decrease or an increase and also a loss of original tendency for production of different products in the parent strain (Behera, 2020). The preferred modification in the *A. niger* genome can be achieved by genetic mutation at the molecular level. In molecular biology, a direct genetic mutation can be done in the specific genes that control fungus biology (Bartnicki-Garcia, 1968; Feofilova, 2010; Li et al., 2016; Muszkieta et al., 2014).

### Chemical and Physical Mutagenesis

The natural process of evolution is not always efficient for immediate implementation in microbial breeding research. Consequently, numerous artificial mutagens have been investigated and employed to enhance frequency of microbial mutations. These include conventional chemical mutagenesis and physical mutagenesis, all aimed at elevating citric acid productivity of microorganisms. Physical and chemical mutagenesis involves interaction of cells with various physical stresses and chemical compounds. This interaction leads to changes in the structure of DNA, which can potentially impact one or more genes (Bhatia et al., 2023). Subsequently, mutated microorganisms are screened and chosen based on desired property after being cultured on a specialized medium.

These mutagens include gamma rays, ultraviolet irradiation, X-rays, alkylating agents like methyl nitroso urea, methyl methanesulfonate, methyl-N-nitro-N-nitrosoguanidine, ethyl methane sulfonate, ethyl nitroso urea, deaminating agents (e.g., hydroxyl amine, nitrous acid), base analogs like 2-aminopurine and 5-bromouracil, and 3-chlorouracil (Bhatia et al. 2023). Javed et al. (2010) used a mutagen source of UV and improved the *A. niger* strain for citric acid yield from 19.4 to 64.2 g/l. Lotfy et al. (2007a) enhanced the *A. niger* strain by 3.2 times compared to the original strain through the application of UV radiation. Mourya et al. (2000) achieved a 130-fold enhancement in citric acid production in the *A. niger* strain by applying the combined effect of UV and ethyl methane sulfonate mutagen.

### Metabolic Engineering

**Fig. 5.**
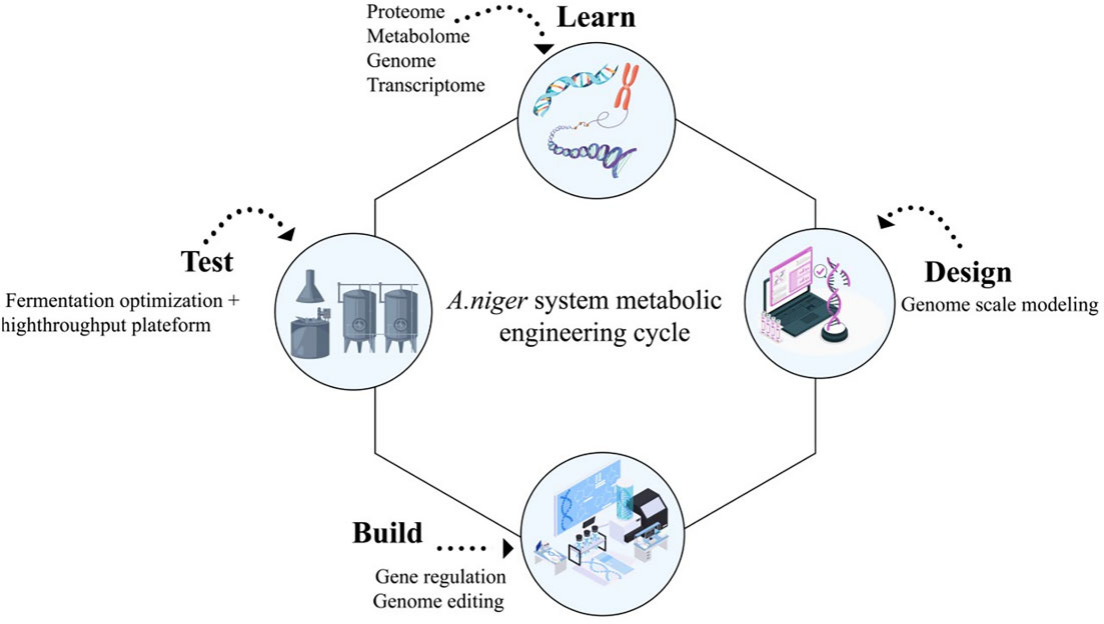
Metabolic engineering cycle.

It has been believed that the most economical and efficient approach involves improving the strain through two main methods: (1) mutagenesis screening strategies and (2) metabolic engineering strategies. Rather than relying on conventional mutagenesis, the latest metabolic engineering has emerged as an influential strategy to enhance citric acid production using any microbe. Metabolic engineering involves intentionally changing cellular metabolism to produce specific compounds, as depicted in Fig. 5. By implementing recombinant DNA technology, it is possible to alter the metabolic pathways of organisms. Citric acid is commonly catabolized by *cis*-aconitase serving as an intermediate of the Krebs cycle. In the glycolysis pathway, ATP and citrate typically exhibit feedback inhibition (Suo et al., 2023). Nevertheless, a lot of citric acid amount is accumulated in *A. niger* due to its active glycolytic pathway. The distinctive regulation of citric acid accumulation in *A. niger* has gained significant attention, and many reviewers have explored the biochemical pathway and mechanisms corresponding to release of the *A. niger* genome (Wu et al., 2022).

Recently, the robust advancement of the CRISPR/Cas9 system has made it feasible to do extremely effective genome-scale genetic modifications in *A. niger*. The CRISPR/Cas system is categorized under two main groups and six major types. Presently, a simpler CRISPR system is the type II CRISPR/Cas9 system and is extensively applied across various species. This type II system is composed of a nuclease (Cas9), *trans*-activating crRNA (tracrRNA), mature CRISPR RNA (crRNA), and RNaseIII. Additionally, crRNA can associate with tracrRNA to form a single-guide RNA (sgRNA) complex (Wu et al., 2022) successfully triggering the Cas9 nuclease to disconnect the mark sequences.

Recently, various CRISPR/Cas9 genome editing systems have been established for *A. niger* (Kim et al., 2018, Savić & Schwank, 2016). A CRISPR/Cas9 system in *Aspergillus* spp is the time reported by Nodvig et al. (2015). Using the RNA polymerase II promoter PgpdA in a single vector, they generated an all-in-one single plasmid system that worked on integration of an sgRNA expression cassette with a Cas9 expression cassette. At 5′ and 3′ ends of the sgRNA were embedded with two ribozymes to ensure its maturation.

Additionally, Kuivanen et al. (2019) co-transformed the sgRNA into protoplasts combined with a plasmid expressing Cas9, considering in vitro transcription by T7 promoters for sgRNA synthesis. While genome editing could be performed instantly with this approach, the stability and absorption of the sgRNA had a strong influence on its effectiveness (Yue et al., 2021).

Furthermore, this method is inappropriate for scenarios requiring conditional or steady-state sgRNA expression, including gene deletion, transcriptional interference, and CRISPR-AID-mediated transcriptional activation (Bendixen et al., 2023). Zheng et al. (2007) discovered an endogenous U6 promoter (PanU6) and used it together with two additional heterologous U6 promoters (PhU6 and PyU6) to assess the gene disruption efficacy of the CRISPR/Cas9 system to address the lack of a U6 promoter in *A. niger*. However, previous studies reported U6 promoters as weak promotors with poor efficiency to guide RNA transcription and gene disruption.

Zheng et al. (2007) established a new CRISPR/Cas9 system that improves sgRNA production by leveraging the 5S rRNA gene. Using short (40 bp) homologous donor DNA, this technique developed hundreds of transformants and significantly increased efficiency, resulting in 100% precision in gene modification. This technique has shown effective in designing chromosomes; multiplex gene insertion and big DNA fragment deletion have made a chassis with reduced mycotoxin levels. The highly effective CRISPR/Cas9 system enables large-scale, high-throughput genome manipulations in *A. niger*, which speeds up the metabolic engineering cycle of the organism.

### Factors Affecting Citric Acid Production

Factors affecting citric acid production are categorized as operational and chemical factors. Different studies were carried out for optimization of components necessary for the fermentation process. In addition, yield of citric acid is highly dependent on media composition, also known as chemical parameters. Currie worked on composition of fermentation media (Currie, 1917). He explored that some components of media are required in large amounts (e.g., sugar, oxygen, and proton), some in minimal amounts (e.g., phosphorus and nitrogen), and others in very minute amounts (e.g., trace elements and manganese).

### Chemical Parameters

#### Carbon source

Much research work has been performed to find the best carbon source for citric acid fermentation. High concentrations of sugar are used for citric acid production in the presence of fungal pellets. Higher sugar concentrations prevent α-ketoglutarate dehydrogenase activity, which ultimately disturbs the Krebs cycle but increases the yield of citric acid (Abonama et al., 2014). Only those sugars are considered best for fermentation, which can be easily broken down by microorganisms and can yield a high amount of citric acid (Mattey, 1992). Many scientists have reported that sucrose is the most favorable carbon source for citric acid production when compared to glucose, fructose, and lactose. This preference stems from invertase’s rapid hydrolysis of sucrose under low pH conditions (Gupta et al., 1976; Hossain et al., 1984; Kubicek et al., 2016; Kubicek-Pranz et al., 1990; Xie & West, 2009). Because of its low molecular weight, sucrose is easily transported into the cell walls of microorganisms and is conveniently available for hydrolysis by intracellular enzymes (El-Holi & Al-Delaimy, 2003). Xylose, starch, sorbitol, arabinose, and pyruvic acid have been used as carbon sources but found not to be good carbon sources for citric acid production because they inhibit fungal development and reduce the accumulation of citric acid (Gil, 2014).

Carbon concentration in the fermentation process acts as a crucial nutrient in citric acid production and should be present in a specific range (e.g., if present in very small amounts, size of my mycelia decreases and it also affects their shapes and morphology) (Papagianni et al., 1999). According to scientific research, the sugar concentration for the production of citric acid is between 10% and 14% (Xu et al., 1989). The production of citric acid drops to zero when the medium has sucrose below 2.5%. High carbon concentration reduces concentration of α-ketoglutarate dehydrogenase, which is an essential enzyme in citric acid production. Several scientists have reported that sugar concentration should be in the range of 14%–22% (Hossain et al., 1984). Honecker et al. (1989) observed that immobilized *A. niger* cells require a less sugar concentration. Sucrose and glucose are not economical carbon sources for the industrial-scale production of citric acid. These carbon sources are expensive compared to other carbon sources. Sugar and beet molasses are good substrates for fermentation (Cevrimli et al., 2009; Ikram-Ul et al., 2004).

#### Nitrogen source

Both for fungal growth and citric acid production, nitrogen is a vital nutrient. Urea, peptone, malt extract, and ammonium salts such as ammonium sulfate and nitrate are used as a source of nitrogen (Grewal & Kalra, 1995). As nitrogen is a part of the proteins, it plays a critical function in fungus growth (Ali et al., 2002). Among salts, ammonium sulfate is preferable as it inhibits production of oxalic acid, which decreases the pH of production media with time. Since molasses is already rich in nitrogen, adding additional nitrogen sources is not necessary whenever using it as a carbon source. Nitrogen concentration should not be more than 0.25%; otherwise, it promotes oxalic acid yield (Gupta et al., 1976; Hang et al., 1977) and decreases citric acid production. Scientists demonstrated optimum levels of nitrogen between 0.1 and 0.4 g/l for citric acid production (Kubicek et al., 2016; Soccol et al., 2006). A high level of nitrogen not only boosts fungal growth but also reduces citric acid yield. Nitrogen plays a crucial role during metabolism, and when the nitrogen level drops below the limiting level, citric acid production starts (Ramesh & Kalaiselvam, 2011).

#### Magnesium source

The source of magnesium promotes the growth of *A. niger* by interfering with morphology of *A. niger*. The magnesium source (e.g., MgSO_4_·5H_2_O) provides free Mg^+2^ in a medium, which is not only valuable for the growth of *A. niger* mycelia but also prevents harmful effects of Fe^+2^. Iron enhances the activity of mitochondrial aconitase because the aconitase enzyme catalyzes the transformation of citrate to isocitrate and reduces the accumulation of citric acid. On the other hand, the above-mentioned activity can be decreased when iron chelator desferrioxamine is added. Iron has a positive effect on Krebs cycle enzymes such as citrate synthase, isocratic dehydrogenase, and succinate dehydrogenase, while its activity decreases when an iron chelator dehydrogenase is added (Sanjay & Sharma, 1994). In this situation, magnesium plays an important role in avoiding such effects. It has been reported that pellets of *A. niger* with the right size, shape, and morphology have been shown to boost the yield of citric acid (Sanjay & Sharma, 1994). Magnesium ions make complex bonds with free ions of Fe^+2^ that precipitate later in the fermentation process (Shoukat et al., 1997). Magnesium is needed for growth and production of citric acid. Kappoor et al. (1982) suggested an optimum magnesium sulfate concentration between 0.02% and 0.025% for citric acid production.

#### Phosphorus source

For fungal growth and citric acid production, phosphate is also very crucial. An essential constituent of DNA and RNA structures is phosphate (Max et al., 2010). When phosphate is used at the optimum concentration, it might have detrimental effects on the production of citric acid, such as the production of other sugar acids such as gluconic, glucuronic, and saccharic acid (Ozdal & Kurbanoglu, 2019). It has been reported that KH_2_PO_4_ concentration would be optimum for fermentation in the range of 0.5–5 g/l (Shu & Johnson, 1948). It has been perceived that citric acid yield increases under limited concentrations of phosphate (Kubicek & Röhr, 1977). Scientists also analyzed favorable aspects and impact on production of citric acid in the presence of a limited source of phosphate concentration (Kubicek & Röhr, 1977; Ozdal & Kurbanoglu, 2019).

### Trace Elements

Heavy metals and trace elements including Cu, Mn, Zn, and Fe are frequently used in fermentation processes. These trace elements should be present in media for growth of *A. niger* and citric acid production (Mattey, 1992; Shu & Johnson, 1948). Manganese is used as a trace element. It is used in a very minute amount, approximately below or equal to 5 μg l^−1^ but sporulation and transporters need to function properly (Shu & Johnson, 1948; Tomlinson and Campbell, 1950). Scientists demonstrated that the rate of citric acid production decreases by half after adding Mn^+2^ in concentration up to 10 mg l^−1^ (Mattey & Bowes, 1978). Tomlinson et al. (1950) reported that ideal values for Fe and Zn are 1.3 and 0.3 ppm, respectively. Rohr (1983) found that the presence of Cu^+2^ enhanced the performance of Fe^+2^ at peak levels, thus increasing the yield of citric acid in the Krebs cycle. Further, a high concentration of Cu^+2^ can demolish lethal effects of Fe^+2^. Benuzzi and Segovia (1996) pointed out that a proper concentration of Cu is necessary to build up the structure of mycelia, which closely affects cell physiology to enhance the yield of citric acid.

### Alcohols Concentration

The citric acid production rate speeds up in the presence of methanol. The permeability of the membrane can be improved by the addition of alcohol into media as alcohol alters the composition of phospholipids. The type of microorganisms and media composition affect the amount of alcohol required for citric acid production (Moyer, 1953). According to Ingrain and Buttke (1984), citric acid yield increases by adding alcohols, which affect the growth of microorganisms and sporulation by changing lipid composition of the membrane. Moyer (1953) observed that the activity of aconitase decreases with the addition of ethanol and increases two times the activity of citrate synthase, which is associated with an increase in citric acid concentration. The concentration of alcohol should be in the range of 1%–5% to inhibit the negative impacts of metal ions (Mn^+2^, Fe^+2^) that decrease citric acid production. Hence, El-Gamal et al. (2018) reported an optimal methanol concentration of 1% for enhanced citric acid production using the yeast strain *C. parapsilosis* NH-3.

### Operational Parameters

#### pH

Media pH is a critical parameter at initial and end stages of the fermentation process, as it directly impacts the growth and metabolic functions of microorganisms accountable for citric acid production, like *A. niger*. Enzymes such as aconitase, citrate synthase, and isocitrate dehydrogenase display their peak activity in the optimal pH range. Metabolic activities of microorganisms such as *A. niger* change the pH of fermentation media due to release of different organic acids such as citric acid that drops the pH. To initiate the fermentation process, pH should be higher than 5 for the proper germination of *A. niger* spores; later on, spores utilize ammonia present in media and release proton that helps in high production of citric acid (Takatsuji & Yoshida, 1998). Researchers Berry et al. (1977) found that a pH of 6.0–7.5 is best for molasses substrate. In contrast, a pH of 3.0 is optimal for the utilization of beet molasses substrate (Roukas & Alichanidis, 1991). During the harvest step of the fermentation process, pH should be less than 2, which is useful for inhibiting the production of side products (e.g., oxalic acid and gluconic acid), which facilitates citric acid recovery (Max et al., 2010).

#### Temperature

An essential parameter in the production of citric acid is temperature (Kappoor et al., 1982). Temperature has a strong effect on the activities of some enzymes, including aconitase, acetyl-CoA, and coenzyme A, which are involved in the Krebs cycle. Beyond specific optimum temperature, enzymes do not work efficiently or denature because enzymes are made of proteins (Prescott & Dunn, 1959). Prescott et al. reported that 25–30^°^C is the best incubation temperature both for the fast production rate and the high yield of citric acid. Above this temperature, oxalic acid production increases. Below this temperature, the growth of organisms slows down (Prescott & Dunn, 1959). In submerged fermentation, temperature can be easily regulated in comparison to solid-state fermentation (Kappoor et al., 1982).

#### Aeration

An aerobic fermentation process proceeds for the production of citric acid, which needs oxygen as a mandatory component. A high oxygen rate has a prominent influence on citric acid yield and process time (Soccol et al., 2006). Productivity of citric acid is highly influenced by dissolved oxygen concentration. High yield must maintain oxygen amounts higher than 25% saturation. The threshold oxygen value is 9%–12% and 12%–13% for growth and production phases, respectively, and the term “threshold oxygen” in microorganisms pertains to minimal oxygen concentration necessary for the growth and viability of a specific microbe (Grewal & Kalra, 1995). Therefore, throughout the production phase, smaller and more compact pellets of *A. niger* mycelia are preferred. When an organism moves toward filament size, the oxygen level suddenly drops to less than 50% of the original value (Soccol et al., 2006). An optimum aeration rate should be implemented to avoid foam formation during the growth phase of *A. niger*. Hence, a low aeration rate is used at start-up stage of the process (Vandenberghe et al., 1999). Anyhow, a very high rate of aeration decreases the partial pressure of dissolved CO_2_ in media. Because CO_2_ is necessary to maintain the partial pressure of oxygen for the proper growth of *A. niger*, an optimum amount of CO_2_ positively affects the yield of citric acid (Vandenberghe, 2000).

### Recovery and Citric Acid Purification

**Fig. 6.**
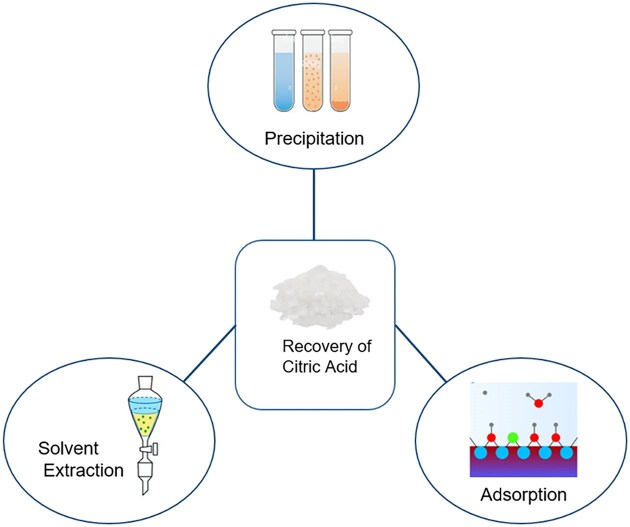
Classification of different citric acid recovery methods.

Citric acid is recovered and purified from fermentation broth by using different techniques, such as adsorption, solvent extraction, and precipitation (Fig. 6). It is significant to choose an economical and efficient procedure that generates less disposal waste after recovery of citric acid, as compared in Table 2.

**Table 2.** Comparison of Precipitation, Solvent Extraction, and Adsorption

Factors	Precipitation	Solvent Extraction	Adsorption
Key parameters	pH, temperature, additives	Solvent choice, pH, mixing	Adsorbent type, pH, contact time
Principle	Formation of solid crystals from solution	Transfer of solute between two liquids	Attachment of solute to solid surface
Recovery efficiency	High to moderate	Moderate to high	Variable, depending on adsorbent
Selectivity	Variable	Variable	High selectivity possible
Ease of operation	Generally simple, depending on process	Requires solvent, mixing, separation	Relatively simple
Purity of product	Can achieve high purity	Moderate purity may require refining	Can achieve high purity
Equipment	Simple equipment	Requires liquid–liquid extraction setup	Adsorption column, packed bed, or batch
Scalability	Generally scalable	Scalable	Scalable

### Precipitation

Precipitation is a phenomenon where a dissolved substance transforms into an insoluble solid state either by interaction of two salts or manipulation of temperature to impact the compound’s solubility. However, the classical method (precipitation) is largely used for recovery of citric acid at commercial scale (Pazouki & Panda, 1998). Several scientists explored classical methods to optimize operational parameters (Heding & Gupta, 1975; Karklin et al., 1984; Pazouki & Panda, 1998) and concentration of CaCl_2_, Ca(OH)_2_, and H_2_SO_4_. The best optimal operational parameters for precipitation of calcium citrate are 50°C temperature for 20 min (Heding & Gupta, 1975). Pazouki and Panda (1998) further reported that washing precipitates several times with water and treating with H_2_SO_4_ to form precipitates of calcium sulfates. After removal of calcium sulfates, the remaining liquid is called mother liquor. The mother liquor needs to be kept at a low temperature for crystal formation of citric acid.

Furthermore, Karklins et al. (1984) precipitated calcium citrate using Ca(OH)_2_, CaCl_2_, and calcium acetate by adjusting the pH between 6.1 and 7.5. Among Ca(OH)_2_, CaCl_2_, and calcium acetate, CaCl_2_ produced the highest quantity of calcium citrate. Calcium citrate was washed out with hot water and dried at 90 and 105°C. Karklin et al. (1984). produced citric acid through fermentation and they obtained a citric acid yield that varied from 64.8% to 92.9% and recovered citric acid from calcium citrate using cation exchange KU-2 using ion exchange principle (Pazouki & Panda, 1998). Pazouki and Panda (1998) have done precipitation of calcium citrate by treating fermentation broth with lime adjusting the pH to 4.3, and after that heating with steam to achieve 60^°^C temperature. Filtration was employed to separate the precipitates, which were then washed out with hot water at 60°C. They further treated with H_2_SO_4_ adjusting the pH at 1.86 to recover citric acid. After treatment with H_2_SO_4_, a large amount of gypsum will be produced. However, a drawback of the classical method is the massive quantity of gypsum and wastewater produced for every ton of citric acid produced.

### Solvent Extraction

The replacement of the classical method is solvent extraction (Wennersten, 1983). Solvent extraction has some benefits over precipitation (e.g., not producing large solid waste gypsum, avoiding sulfuric acid and lime addition, and preventing the formation of CaSO_4_) (Grewal & Kalra, 1995). Scientists have developed a solvent extraction method for citric acid recovery using a different type of solvent or combination of solvents. Yi et al. (1987) used a combination of solvents containing 60%–70% N, N-disubstituted alkyl amide, and 30%–40% butylacetate and achieved 96.6% pure citric acid. They used a countercurrent extraction column containing five stages. Activated carbon and NaCl were used to inhibit emulsification (Pazouki & Panda, 1998). Amine extraction has been developed by several authors to recover carboxylic acids from the aqueous phase (Pazouki & Panda, 1998; Soccol & Vandenberghe, 2003).

Amine extraction technique is well-known for recovery of citric acid. Amine extraction has proved a good prospect for citric acid recovery, as a nontoxic solvent is the main demand of this process. Primary, secondary, tertiary, and quaternary amines have a good distribution coefficient. Primary amine has a good distribution coefficient but also has a good affinity for water. As distribution coefficient is a parameter within solvent extraction procedures, aiming to measure how a solute is distributed or divided between two phases that cannot be mixed, usually involving a solvent phase and an aqueous phase. Secondary amines produce irreversible amide formation followed by distillation. However, with increasing loading of quaternary amines, the ability to build stable emulsion is also increased. Tertiary amines are a good option for amine extraction as a solvent because tertiary amines have a large number of R groups for extraction of acids as compared to other amines (Pazouki & Panda, 1998).

### Adsorption

Adsorption is a phenomenon where molecules, ions, or particles within a fluid, such as gas or liquid, attach or gather on the surface of a solid material or interface. This adherence is driven by attractive forces existing between substance being adsorbed (known as adsorbate) and solid material or interface (referred to as the adsorbent) (Pazouki & Panda, 1998). Using ion exchange resins, many researchers investigated the recovery and purification of organic acid (such as citric acid oxalic acid) and sugar. Maria Papagianni (2007) conducted innovative research on the adsorption (ion exchange process) of several acids, including lactic acid, malic acid, acetic acid, and another organic acid on a profitable resin Diaion WA30. Carlos Jacinto et al. (2020) also worked on adsorption of citric acid using different adsorbents such as Amberlite IRA-900(Cl), IRA-67, polyvinylpyrrolidone (PVP), retardion 11A8, and polyvinylpolypyrrolidone (PVPP). They have got good absorption capacity for citric acid in PVP adsorbent with adsorption capacity (>30 wt. %) at low pH.

Further, Delgado Dobladez et al. (2019) reported recovery of citric acid above 95% having a purity above 99%. They have used a simulated moving bed column containing a Reillex® 425 resin worked as an adsorbent and desorption of adsorbed material is performed with methanol. Each recovery technique has advantages and disadvantages; still, the classical method is more popular among all these techniques because of its low operational cost. Carlos Jacinto et al. (2020) developed an adsorption process for the citric acid recovery using Dowex™ Marathon™ WBA resin and recovered 85% of citric acid with a purity of 92% at low pH ≈1.5. This resin has shown a good absorption capacity of 0.46 g citric acid/g of resin used.

### Applications

**Fig. 7.**
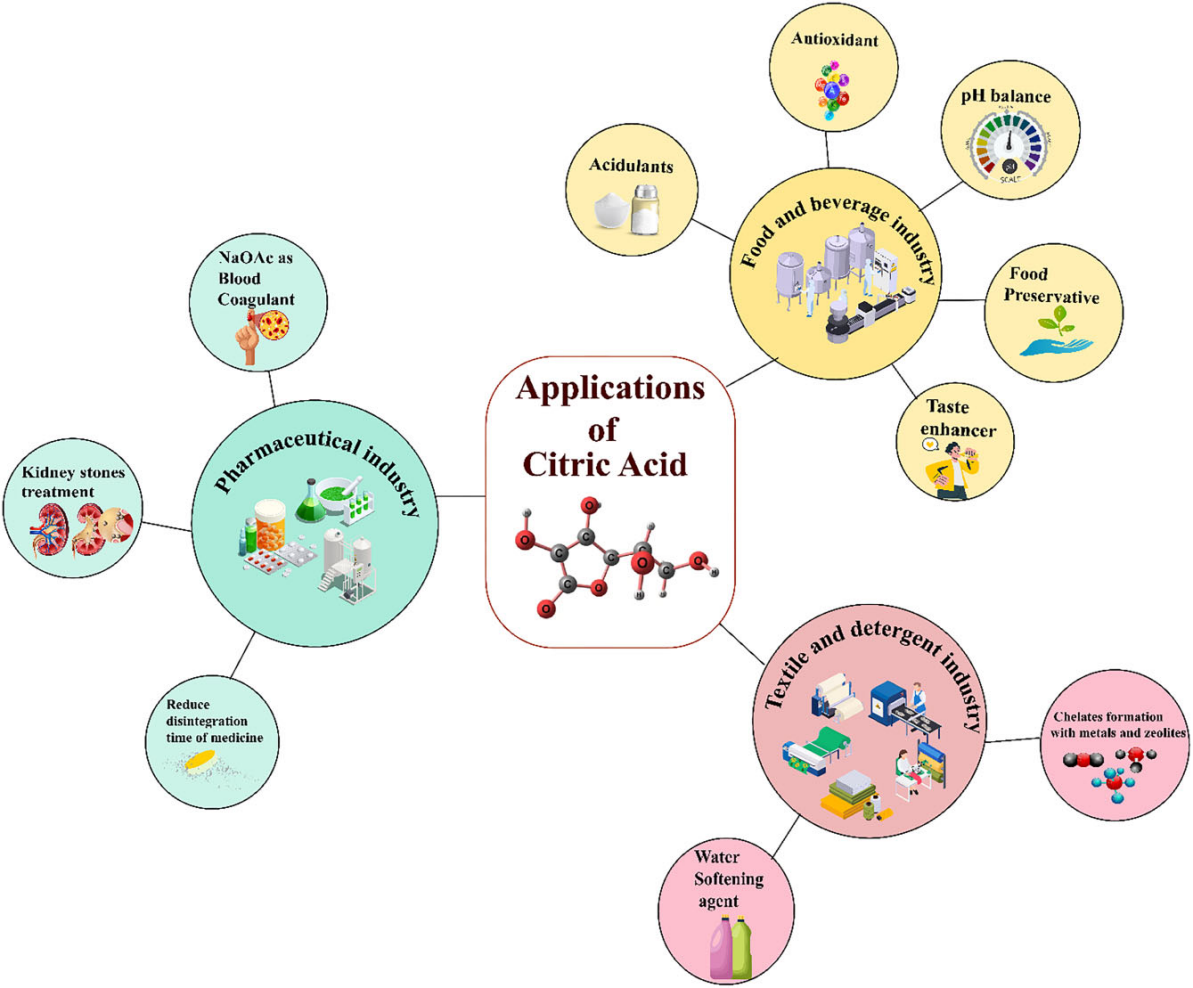
Applications of citric acid in different industries.

Citric acid has numerous applications across various industries, particularly in food production due to its characteristic sour taste. The WHO expert/Joint FAO committee approved it as a “GRAS” worldwide (Pandey et al., 2001; Soccol & Vandenberghe, 2003; Vandenberghe et al., 1999). It is predominantly applied in food, pharmaceutical, and cosmetics industries (Fig. 7). With the expansion in processed food consumption, citric acid demand is rising with growing population (Mores et al., 2021). According to a new survey in 2016, 62.53% of global citric acid is consumed in food and beverages industries. Further, personal care and pharmaceuticals are utilizing 17.08% of citric acid. However, 12.27% and 8.12% of total citric acid are consumed in detergents and other applications, respectively. On the other hand, citric acid demand has increased in food and beverages, approximately 6.03% from 2016 to 2021, and for other industries, 5.084% for the personal care and pharmaceutical industry. Up until 2025, the consumption rate of citric acid has the potential to increase demand at a rate of 5.24% (Mores et al., 2021). An assessment of the worldwide citric acid market’s dimensions stood at approximately $3.52 billion in 2022. According to Zhang et al. (2023), the economy will grow at a compound annual growth rate of 3.82% within the predicted timeframe from 2023 to 2032, valued at around $5.12 billion.

### Pharmaceuticals

Citric acid can perform multiple functions due to presence of three carboxyl and one hydroxyl groups as well as an economical and nontoxic monomer for the pharmaceutical industry (Salihu et al., 2021). Citric acid plays a critical part in controlling phase of mineralization, metabolism, stone prevention, and neuronal excitations (Ma et al., 2018). Trisodium citrate constitutes one of the tribasic salts of citric acid that comes in both dihydrous and anhydrous forms. It is widely used to cure kidney stones. The dihydrous form has taken up fluids and aptitude to take up other fluids and water (Lambros et al., 2022). Sodium citrate is used for storage of blood acting as an anticoagulant. It is the main component of oral rehydration solution (Nangare et al., 2021). The presence of citric acid in formulation of different medicines has solved many problems because of its chelating characteristics (Salihu et al., 2021). Furthermore, citric acid shows amazing fluorescence in many biomedical activities (Nangare et al., 2021). Citric acid minimized the disintegration time of tablets getting benefit of its hydrophilic property. Additionally, it presents efficient drug delivery and loading to targeted areas (Lambros et al., 2022; Nangare et al., 2021). People use various products containing citric acid as it improves magnesium, phosphorous, and calcium absorption via the gut. Moreover, it prevents calcium crystal formation in the kidney due to mineral-binding characteristics (Salihu et al., 2021).

### Food and Beverages

Citric acid is an essential product for food and beverages industry. Two-thirds of global citric acid is consumed by the food and beverages industry, as it performs many diverse functions such as preserving food by inhibiting the growth of yeast, mold, fungi, and bacteria. It improves food taste due to its sour taste (Behera et al. 2021). Moreover, citric acid is ordinarily applied as a flavor enhancer, additive, antioxidant, and pH adjustment. It is used in carbonated soft drinks as an acidulant (Mores et al. 2023). Citric acid has a tendency to form chelates and pH adjustments. Therefore, it is used in food industry for frozen food items to prevent color and flavor alteration and also enhance the shelf life of frozen fish (Moledina et al. 1977).

### Textile and Detergents

Citric acid is gaining popularity in the detergent industry by providing less harmful formulation benefits as compared to phosphates by providing good chelating properties. In addition, it provides benefits for co-builders with zeolites in liquid detergents (Mores et al., 2023). On the other hand, it is also used as a household dishwasher and detergent because it chelates metals such as Mg^+2^ and Ca^+2^ present in hard water. Ultimately, it removes water hardness and helps out in making foam. Detergents perform well in soft water (Ciriminna et al., 2017). Citrate is applied in the textile industry as a foaming agent to enhance the suppleness of fabrics (Hu et al., 2019). When metals combine with citric acid, they tend to form chelates, making them water soluble. Hence, it is used for cleaning boilers and removing scaling from various equipment, placed at power stations (Behera, 2020). Furthermore, it is used for cleaning nuclear places that are contaminated with radionuclear particles (Kantar & Honeyman, 2006).

### Agriculture

The application of low-molecular-weight organic acid such as citric acid is crucial for altering the bioavailability and chemical reactivity of the heavy metals and improving phytoextraction. Citric acid is widely used as a synthetic chelator for improving phytoextraction ultimately enhancing plant growth (Han et al., 2018). Spraying of a combination of citric acid with some micronutrients (Zn, Mn, and Fe) named citrine on the maize plants resulted in wonderful growth, high yield, and productivity of seeds with amazing quality (Larqué et al., 2010; Salas Pérez et al., 2018). Citric acid facilitates degrade-conjugated phenols into simpler phenolic compounds and, as a result, promotes the synthesis of phenylpropanoid-derived compounds and activates signaling pathways that enhance antioxidant activity in plants. The advantageous impact of antioxidants considering citric acid can be attributed to its major contribution in increasing cell division, and protecting the cell wall from free radicals, which play a crucial role in plant senescence. Furthermore, these properties are closely linked to their tendency to mitigate stress caused by diseases, salinity, and drought. Additionally, citric acid demonstrates auxinic activity, thereby encouraging plant growth and stimulating the synthesis of nutraceutical compounds, such as phenolic compounds and flavonoids, which work as antioxidants (Elade, 1992; Pérez‐Balibrea, 2008; Raskin, 1992).

### Cosmetics

In cosmetics, citric acid mainly acts as an antioxidant and exfoliating agent. Citric acid mitigates the influence of free radical damage on the skin although contributing to skin toning and firmness. When combined with citrate, citric acid demonstrates exceptional buffering capacity, although its superior metal ion chelating properties enhance its physicochemical properties, making it highly suitable for wonderful applications in the cosmetics industry. An efficient buffering capacity property is very helpful for improving and maintaining the stability of the products (Ciriminna et al., 2017). In 2011, Rothenberg and Alberts from the University of Amsterdam found a unique application of citric acid as a cross-linking agent. They revealed that citric acid and glycerol do polymerize reactions to form a water-soluble thermoset resin, presenting quick degradation in an environment (Alberts & Rothenberg., 2012).

## Conclusion and Future Outlook

This review extensively explores the biochemistry, bioengineering, and metabolic engineering techniques involved in citric acid production and its hyperproduction processes. Given all the above-mentioned aspects, it becomes clear that producing citric acid industrially at a reasonable cost is a very complicated and sensitive procedure. It relies on several variables, including the raw materials used, the microorganisms chosen, the fermentation process, appropriate design of biofermenter with specific control over operational parameters, the metabolic pathways and factors influencing the production of citric acid, additionally methods for strain improvement, quantification, and recovery. Substantial research efforts are essential to establish solutions for these incoming challenges.

The global citric acid market observed significant growth throughout the COVID-19 pandemic, driven by the highest demand according to the rising number of individuals affected by the COVID-19 virus. There has been observed notable attention in utilizing bulk amounts of citric acid in the manufacturing of pharmaceuticals, food and beverages, and cosmetics. Furthermore, citric acid has antimicrobial properties that lead to faster production of disinfectant products. This peak demand for citric acid has particularly encouraged manufacturing of disinfectant items based on citric acid, such as germicidal detergent wipes, comet disinfecting bathroom cleaner, and other solutions targeted to preventing COVID-19 infections. Additionally, there is a rising trend of consuming food and beverages comprising citric acid to enhance individual immunity and combat COVID-19 infections. It will be compulsory to closely monitor changes in consumer preferences, technology advancements, and regulatory developments due to the increased demand for citric acid in recent years.

## Data Availability

The data underlying this article are available in the article and in its online supplementary material (if any).

## References

[bib1] Abonama O., Mahrous E. H., Hamza H. (2014). Production of citric acid by *Candida lipolytica* under fermentation conditions using a Plackett-Burman design. American Journal of Food and Nutrition, 2(3), 43–48.

[bib2] Agnihotri V. P. (1966). Studies on aspergilli xix. Role of carbon and nitrogen on citric acid production. Mycopathologia et Mycologia Applicata, 30(2), 115–120. 10.1007/bf02130357 5973499

[bib3] Ahmed S., Smith J., Anderson J. (1972). Mitochondrial activity during citric acid production by *Aspergillus niger*. Transactions of the British Mycological Society, 59(1), 51–61.

[bib4] Alberts A. H., Rothenberg G. (2012). Process for preparing foamed polymer. WO2012052385.

[bib5] Ali S., Haq I.-U., Qadeer M., Iqbal J. (2002). Production of citric acid by *Aspergillus niger* using cane molasses in a stirred fermentor. Electronic Journal of Biotechnology, 5(3), 19–20.

[bib6] Arora S., Rani R., Ghosh S. (2018). Bioreactors in solid state fermentation technology: Design, applications and engineering aspects. Journal of Biotechnology, 269, 16–34. 29408199 10.1016/j.jbiotec.2018.01.010

[bib7] Ballardo C., del C., Vargas-García M., Sánchez A., Barrena R., Artola A. (2020). Adding value to home compost: Biopesticide properties through *Bacillus thuringiensis* inoculation. Waste Management, 106, 32–43. 32179419 10.1016/j.wasman.2020.03.003

[bib8] Bartnicki-Garcia S. (1968). Cell wall chemistry, morphogenesis, and taxonomy of fungi. Annual Review of Microbiology, 22(1), 87–108. 10.1146/annurev.mi.22.100168.000511 4879523

[bib9] Behera B. C. (2020). Citric acid from *Aspergillus niger*: A comprehensive overview. Critical Reviews in Microbiology, 46(6), 727–749. 33044884 10.1080/1040841X.2020.1828815

[bib10] Behera B. C., Mishra R., Mohapatra S. (2021). Microbial citric acid: Production, properties, application, and future perspectives. Food Frontiers, 2(1), 62–76.

[bib11] Bendixen L., Jensen T. I., Bak R. O. (2023). CRISPR-Cas-mediated transcriptional modulation: The therapeutic promises of CRISPRa and CRISPRi. Molecular Therapy, 31(7), 1920–1937. 36964659 10.1016/j.ymthe.2023.03.024 PMC10362391

[bib12] Benghazi L., Record E., Suárez A., Gomez-Vidal J. A., Martínez J., de La Rubia T. (2014). Production of the phanerochaete flavido-alba laccase in *Aspergillus niger* for synthetic dyes decolorization and biotransformation. World Journal of Microbiology and Biotechnology, 30(1), 201–211. 23884844 10.1007/s11274-013-1440-z

[bib13] Benuzzi D. A., Segovia R. F. (1996). Effect of the copper concentration on citric acid productivity by an *Aspergillus niger* strain. Applied Biochemistry and Biotechnology (USA), 61(3),393–397. 10.1007/BF02787810 9100359

[bib14] Berovic M., Legisa M. (2007). Citric acid production. Biotechnology Annual Review, 13, 303–343. 10.1016/S1387-2656(07)13011-8 17875481

[bib15] Berry D., Chmiel A., Al Obaidi Z. (1977). Citric acid production by *Aspergillus niger*. Genetics and Physiology of Aspergillus, 1, 405–426.

[bib16] Bhatia S. K., Kumar V., Kumar V., Bhatia R. K., Yang Y.-H. (2023). Microbial activity and productivity enhancement strategies. In A. K. (Ed.), Basic biotechniques for bioprocess and bioentrepreneurship (pp. 85–104.). Elsevier.

[bib17] Carboué Q., Rébufa C., Dupuy N., Roussos S., Bombarda I. (2019). Solid state fermentation pilot-scaled plug flow bioreactor, using partial least square regression to predict the residence time in a semicontinuous process. Biochemical Engineering Journal, 149, 107248.

[bib18] Cevrimli B. S., Kariptas E., Ciftci H. (2009). Effects of fermentation conditions on citric acid production from beet molasses by *Aspergillus niger*. Asian Journal of Chemistry, 21(4), 3211–3218.

[bib19] Chmiel A. (1975). Kinetic studies on citric acid production by *Aspergillus niger*. II. The two-stage process. Acta Microbiologica Polonica Series B, 7(4), 237–242. 5858

[bib20] Ciriminna R., Meneguzzo F., Delisi R., Pagliaro M. (2017). Citric acid: Emerging applications of key biotechnology industrial product. Chemistry Central Journal, 11(1), 1–9. 28326128 10.1186/s13065-017-0251-y PMC5342991

[bib21] Currie J. N. (1917). The citric acid fermentation of *Aspergillus niger*. Journal of Biological Chemistry, 31(1), 15–37.

[bib22] Delgado Dobladez J. A., Águeda Maté V. I., Uribe Santos D. L., Torrellas S. Á., Larriba M. (2019). Citric acid purification by simulated moving bed adsorption with methanol as desorbent. Separation Science and Technology, 54(6), 930–942.

[bib23] Drysdale C., McKay A. (1995). Citric acid production by *Aspergillus niger* in surface culture on inulin. Letters in Applied Microbiology, 20(4), 252–254. 7766122 10.1111/j.1472-765x.1995.tb00440.x

[bib24] Elade Y. (1992). The use of antioxidants to control gray mould (*Botrytis cineria*) and white mould (*Sclerotinia sclerotiorum*) in various crops. Plant Pathology, 41(4), 417–426.

[bib25] El-Gamal M. S., Desouky S. E.-S., Abdel-Rahman M. A., Khattab A.-R. M. (2018). High-temperature citric acid production from sugar cane molasses using a newly isolated thermotolerant yeast strain, *Candida parapsilosis* NH-3. International Journal of Advanced Research in Biological Sciences (IJARBS), 5(7), 187–211.

[bib26] El-Holi M. A., Al-Delaimy S. (2003). Citric acid production from whey with sugars and additives by *Aspergillus niger*. African Journal of Biotechnology, 2(10), 356–359.

[bib27] Feofilova E. (2010). The fungal cell wall: Modern concepts of its composition and biological function. Microbiology (Reading, England), 79(6), 711–720. 21774151

[bib28] Gil P. C. N. (2014). Adição do Ácido Ricinoleico na dieta de equinos. Universidade de São Paulo.

[bib29] González-Sáiz J., Fernández-Torroba M., Pizarro C. (1997). Application of weakly basic copolymer polyacrylamide (acrylamide-CO-N, N′-dimethylaminoethyl methacrylate) gels in the recovery of citric acid. European Polymer Journal, 33(4), 475–485.

[bib30] Grewal H., Kalra K. (1995). Fungal production of citric acid. Biotechnology Advances, 13(2), 209–234. 14537820 10.1016/0734-9750(95)00002-8

[bib31] Gupta J., Heding L., Jorgensen O. (1976). Effect of sugars, hydrogen ion concentration and ammonium nitrate on the formation of citric acid by *Aspergillus niger*. Acta Microbiologica Academiae Scientiarum Hungaricae, 23(1), 63–67. 7101

[bib32] Habison A., Kubicek C. P., Röhr M. (1983). Partial purification and regulatory properties of phosphofructokinase from *Aspergillus niger*. Biochemical Journal, 209(3), 669–676. 6223622 10.1042/bj2090669 PMC1154144

[bib33] Han Y., Zhang L., Gu J., Zhao J., Fu J. (2018). Citric acid and EDTA on the growth, photosynthetic properties and heavy metal accumulation of Iris halophila Pall. cultivated in Pb mine tailings. International Biodeterioration & Biodegradation, 128, 15–21. 10.1016/j.ibiod.2016.05.011

[bib34] Hang Y., Splittstoesser D., Woodams E., Sherman R. (1977). Citric acid fermentation of brewery waste. Journal of Food Science, 42(2), 383–384.

[bib35] Heding L. G., Gupta J. (1975). Improvement of conditions for precipitation of citric acid from fermentation mash. Biotechnology and Bioengineering, 17(9), 1363–1364.

[bib36] Honecker S., Bisping B., Yang Z., Rehm H.-J., (1989). Influence of sucrose concentration and phosphate limitation on citric acid production by immobilized cells of *Aspergillus niger*. Applied Microbiology and Biotechnology, 31(1), 17–24.

[bib37] Hossain M., Brooks J., Maddox I. (1984). The effect of the sugar source on citric acid production by *Aspergillus niger*. Applied Microbiology and Biotechnology, 19(6), 393–397.

[bib38] Hu W., Li W.-j., Yang H.-Q., Chen J.-H. (2019). Current strategies and future prospects for enhancing microbial production of citric acid. Applied Microbiology and Biotechnology, 103(1), 201–209. 30421107 10.1007/s00253-018-9491-6

[bib39] Ikram-Ul H., Ali S., Qadeer M., Iqbal J. (2004). Citric acid production by selected mutants of *Aspergillus niger* from cane molasses. Bioresource Technology, 93(2), 125–130. 15051073 10.1016/j.biortech.2003.10.018

[bib40] Ingrain L., Buttke T. (1984). Effects of alcohols on microorganisms. Advances in Microbial Physiology, 25(25), 300. 10.1016/s0065-2911(08)60294-5 6398622

[bib41] Jacinto C., Ramos E., López D. (2020). Citric acid recovery from a synthetic fermentation broth by ion-exchange resins. Bistua Revista de la Facultad de Ciencias Basicas, 18(2), 9–14.

[bib42] Javed S., Asgher M., Sheikh M. A., Nawaz H. (2010). Strain improvement through UV and chemical mutagenesis for enhanced citric acid production in molasses-based solid state fermentation. Food Biotechnology, 24(2), 165–179.

[bib43] Jernejc K., Cimerman A., Perdih A. (1992). Composition of *Aspergillus niger* mycelium during growth on productive and unproductive substrates. Journal of Biotechnology, 25(3), 341–348.

[bib44] Kantar C., Honeyman B. D. (2006). Citric acid enhanced remediation of soils contaminated with uranium by soil flushing and soil washing. Journal of Environmental Engineering, 132(2), 247–255.

[bib45] Kapilan R. (2015). Solid state fermentation for microbial products: A review. Archives of Applied Science Research, 7(8), 21–25.

[bib46] Kappoor K., Chudhary K., Tauro P. (1982). Citric acid. In G. Reed (Ed.), Prescott and Dunn's industrial microbiology. AVI Publishing Co.

[bib47] Karklin R., Ramina L., Raso R. (1984). Factors affecting the isolation of Ca-citrate from fermentation solution of n-alkanes. Biosint Oksikilot Ketokislot Mikroorg, 1, 43–51.

[bib48] Khurshid S., Ali S., Ashraf H., Qadeer M., Rajoka M. I. (2001). Mutation of *Aspergillus niger* for hyperproduction of citric acid from black strap molasses. World Journal of Microbiology and Biotechnology, 17, 35–37.

[bib49] Kim D., Alptekin B., Budak H. (2018). CRISPR/Cas9 genome editing in wheat. Functional & Integrative Genomics, 18, 31–41. 28918562 10.1007/s10142-017-0572-x

[bib50] Krull R., Cordes C., Horn H., Kampen I., Kwade A., Neu T. R., Nörtemann B. (2010). Morphology of filamentous fungi: Linking cellular biology to process engineering using *Aspergillus niger*. Advances in Biochemical Engineering/Biotechnology, 121,:1–21. 20490972 10.1007/10_2009_60

[bib51] Krull R., Wucherpfennig T., Esfandabadi M. E., Walisko R., Melzer G., Hempel D. C., Kampen I., Kwade A., Wittmann C. (2013). Characterization and control of fungal morphology for improved production performance in biotechnology. Journal of Biotechnology, 163(2), 112–123. 22771505 10.1016/j.jbiotec.2012.06.024

[bib52] Książek E. (2023). Citric acid: Properties, microbial production, and applications in industries. Molecules (Basel, Switzerland), 29(1), 22. 38202605 10.3390/molecules29010022 PMC10779990

[bib54] Kubicek C. (1998). The role of sugar uptake and channeling for citric acid accumulation by *Aspergillus niger*. Food Technology and Biotechnology, 36(3), 173–175.

[bib55] Kubicek C., Röhr M. (1977). Influence of manganese on enzyme synthesis and citric acid accumulation in *Aspergillus niger*. European Journal of Applied Microbiology, 4(3), 167–175.

[bib56] Kubicek L., Milner R., An Q., Kow K., Chang M., Cooke K., Fox L., Farese J., Bacon N., Lurie D. (2016). Outcomes and prognostic factors associated with canine sinonasal tumors treated with curative intent cone-based stereotactic radiosurgery (1999–2013). Veterinary Radiology & Ultrasound, 57(3), 331–340. 26880676 10.1111/vru.12349

[bib57] Kubicek-Pranz E. M., Mozelt M., Rōhr M., Kubicek C. P. (1990). Changes in the concentration of fructose 2,6-bisphosphate in *Aspergillus niger* during stimulation of acidogenesis by elevated sucrose concentration. Biochimica et Biophysica Acta, 1033(3), 250–255. 2156568 10.1016/0304-4165(90)90128-j

[bib58] Kuivanen J., Biz A., Richard P. (2019). Microbial hexuronate catabolism in biotechnology. AMB Express, 9(1), 16. 30701402 10.1186/s13568-019-0737-1 PMC6353982

[bib60] Lambros M., Tran T., Fei Q., Nicolaou M. (2022). Citric acid: A multifunctional pharmaceutical excipient. Pharmaceutics, 14(5), 972. 35631557 10.3390/pharmaceutics14050972 PMC9148065

[bib61] Larqué-Saavedra A., Martín-Mex R., Nexticapan-Garcéz Á., Vergara-Yoisura S., Gutiérrez-Rendón M. (2010). Efecto del ácido salicílico en el crecimiento de plántulas de tomate (*Lycopersicon esculentum* Mill.). Revista Chapingo Serie Horticultura, XVI(3), 183–187.

[bib62] Li M., Jiang C., Wang Q., Zhao Z., Jin Q., Xu J.-R., Liu H. (2016). Evolution and functional insights of different ancestral orthologous clades of chitin synthase genes in the fungal tree of life. Frontiers in Plant Science, 7, 37. 26870058 10.3389/fpls.2016.00037 PMC4734345

[bib63] Lingappa K., Naik C., Babu C. V., Ramakrishna D., Reddy M. V. (2002). Coconut cake: A novel substrate for citric acid production under solid state fermentation. Indian Journal of Microbiology, 42(4), 347–350.

[bib64] Lodhi A. K., Asghar M., Zia M. A., Ambreen S., Asad M. J. (2001). Production of citric acid from waste bread by *Aspergillus niger*. Journal of Biological Sciences, 1(4), 182–183.

[bib65] Lotfy W. A., Ghanem K. M., El-Helow E. R. (2007). Citric acid production by a novel *Aspergillus niger* isolate: II. Optimization of process parameters through statistical experimental designs. Bioresource Technology, 98(18), 3470–3477. 17317159 10.1016/j.biortech.2006.11.032

[bib66] Lotfy W. A., Ghanem K. M., El-Helow E. R. (2007). Citric acid production by a novel *Aspergillus niger* isolate: I. Mutagenesis and cost reduction studies. Bioresource Technology, 98(18), 3464–3469. 17223558 10.1016/j.biortech.2006.11.007

[bib67] Ma C., Gerhard E., Lu D., Yang J. (2018). Citrate chemistry and biology for biomaterials design. Biomaterials, 178, 383–400. 29759730 10.1016/j.biomaterials.2018.05.003 PMC6366999

[bib68] Mattey M. (1992). The production of organic acids. Critical Reviews in Biotechnology, 12(1–2), 87–132. 1733523 10.3109/07388559209069189

[bib69] Mattey M., Bowes I. (1978). Citrate regulation of NADP+-specific isocitrate dehydrogenase of Aspergillus niger. Portland Press Ltd. 10.1042/bst0061224 33856

[bib70] Mattey M., Kristiansen B. (1999). A brief introduction to citric acid biotechnology. In B. Kristiansen, J. Linden, & M. Mattey (Eds.), Citric acid biotechnology. (pp. 1–10).CRC Press

[bib71] Max B., Salgado J. M., Rodríguez N., Cortés S., Converti A., Domínguez J. M. (2010). Biotechnological production of citric acid. Brazilian journal of Microbiology, 41(4), 862–875. 24031566 10.1590/S1517-83822010000400005 PMC3769771

[bib72] Moledina K., Regenstein J., Baker R., Steinkraus K. (1977). Effects of antioxidants and chelators on the stability of frozen stored mechanically deboned flounder meat from racks after filleting. Journal of Food Science, 42(3), 759–764.

[bib73] Mores S., de Souza Vandenberghe L. P., Júnior A. I. M., de Carvalho J. C., de Mello A. F. M., Pandey A., Soccol C. R. (2021). Citric acid bioproduction and downstream processing: Status, opportunities, and challenges. Bioresource Technology, 320, 124426. 33249260 10.1016/j.biortech.2020.124426

[bib74] Mores S., de Souza Vandenberghe L. P., Martinez-Burgos W. J., Rodrigues C., Soccol C. R. (2023). Simultaneous reuse and treatment of sugar-sweetened beverage wastes for citric acid production. Journal of Food Science and Technology, 60:(9), 2401–2407. 37424583 10.1007/s13197-023-05761-9 PMC10326170

[bib75] Mourya S., Jauhri K. S. (2000). Production of citric acid from starch-hydrolysate by *Aspergillus niger*. Microbiological Research, 155(1), 37–44. 10830898 10.1016/S0944-5013(00)80020-8

[bib76] Moyer A. J. (1953). Effect of alcohols on the mycological production of citric acid in surface and submerged culture. Applied Microbiology, 1(1), 1–6. 13008421 10.1128/am.1.1.1-7.1953 PMC1056848

[bib77] Muszkieta L., Aimanianda V., Mellado E., Gribaldo S., Alcàzar-Fuoli L., Szewczyk E., Prevost M. C., Latgé J. P. (2014). Deciphering the role of the chitin synthase families 1 and 2 in the in vivo and in vitro growth of *Aspergillus fumigatus* by multiple gene targeting deletion. Cellular microbiology, 16(12), 1784–1805. 24946720 10.1111/cmi.12326

[bib78] Nangare S., Vispute Y., Tade R., Dugam S., Patil P. (2021). Pharmaceutical applications of citric acid. Future Journal of Pharmaceutical Sciences, 7(1), 1–23.

[bib80] Nelson D. L., Cox M. M. (2017). Lehninger principles of biochemistry (7th ed.) W.H. Freeman.

[bib81] Nødvig C. S., Nielsen J. B., Kogle M. E., Mortensen U. H. (2015). A CRISPR-Cas9 system for genetic engineering of filamentous fungi. PLoS One, 10(7), e0133085. 26177455 10.1371/journal.pone.0133085 PMC4503723

[bib82] Oiza N., Moral-Vico J., Sánchez A., Oviedo E. R., Gea T. (2022). Solid-state fermentation from organic wastes: a new generation of bioproducts. Processes, 10(12), 2675.

[bib83] Ozdal M., Kurbanoglu E. B. (2019). Citric acid production by *Aspergillus nige*r from agro-industrial by-products: Molasses and chicken feather peptone. Waste and Biomass Valorization, 10(3), 631–640.

[bib84] Pallares J., Rodriguez S., Sanroman A. (1996). Citric acid production in submerged and solid state culture of *Aspergillus niger*. Bioprocess Engineering, 15(1), 31–33.

[bib85] Palmieri F. (2013). Transporters in the mitochondria: Unraveling their role in energy metabolism. Journal of Biological Chemistry, 288(19), 11996–12003.

[bib86] Pandey A. (1992). Recent process developments in solid-state fermentation. Process Biochemistry, 27(2), 109–117.

[bib87] Pandey A. (2003). Solid-state fermentation. Biochemical Engineering Journal, 13(2–3), 81–84. 10.1016/s1369-703x(00)00065-6 10908866

[bib88] Pandey A., Soccol C. R., Rodriguez-Leon J. A., Nigam P. S.-N. (2001). Solid state fermentation in biotechnology: Fundamentals and applications. Asiatech Publishers Inc.

[bib89] Papagianni M. (2007). Advances in citric acid fermentation by *Aspergillus niger*: Biochemical aspects, membrane transport and modeling. Biotechnology Advances, 25(3), 244–263. 17337335 10.1016/j.biotechadv.2007.01.002

[bib90] Papagianni M. (2017). Microbial bioprocesses. In A. Pandey, S. Negi, & C. R. Soccol (Eds.),​ ​​​​​​Current developments in biotechnology and bioengineering (pp. 45–72.). Elsevier.

[bib91] Papagianni M., Mattey M., Berovic M., Kristiansen B. (1999). *Aspergillus niger* morphology and citric acid production in submerged batch fermentation: Effects of culture pH, phosphate and manganese levels. Food Technology and Biotechnology, 37, 165–172.

[bib94] Pazouki M., Felse P., Sinha J., Panda T. (2000). Comparative studies on citric acid production by *Aspergillus niger* and *Candida lipolytica* using molasses and glucose. Bioprocess engineering, 22(4), 353–361.

[bib95] Pazouki M., Panda T. (1998). Recovery of citric acid—a review. Bioprocess Engineering, 19(6), 435–439.

[bib96] Pérez-Balibrea S., Moreno D. A., García-Viguera C. (2008). Influence of light on health-promoting phytochemicals of broccoli sprouts. Journal of the Science of Food and Agriculture, 88(5), 904–910.

[bib97] Prescott S., Dunn C. (1959). The citric acid fermentation. Industrial Microbiology, 3, 533–577.

[bib98] Ramesh T., Kalaiselvam M. (2011). An experimental study on citric acid production by *Aspergillus niger* using *Gelidiella acerosa* as a substrate. Indian Journal of Microbiology, 51(3), 289–293. 22754005 10.1007/s12088-011-0066-9 PMC3209904

[bib99] Raskin I. (1992). Salicylate, a new plant hormone. Plant Physiology, 99(3), 799. 16669002 10.1104/pp.99.3.799 PMC1080546

[bib100] Ratledge C. (2001). Biochemistry and physiology of growth and metabolism. In C. Ratledge & B. Kristiansen (Eds.), Basic biotechnology. Cambridge University Press.

[bib101] Rodrigues C., de Souza Vandenberghe L. P., Teodoro J., Pandey A., Soccol C. R. (2009). Improvement on citric acid production in solid-state fermentation by *Aspergillus niger* LPB BC mutant using citric pulp. Applied Biochemistry and Biotechnology, 158(1), 72–87. 18925364 10.1007/s12010-008-8370-5

[bib102] Rohr M. (1983). Citric acid. Biotechnology (Reading, Mass.), 3, 419–454.

[bib103] Roukas T., Alichanidis E. (1991). Citric acid production from beet molasses by cell recycle of *Aspergillus niger*. Journal of Industrial Microbiology, 7(1), 71–73.

[bib104] Rubio M. C., Maldonado M. C. (1995). Purification and characterization of invertase from *Aspergillus niger*. Current Microbiology, 31(2), 80–83.

[bib105] Salas-Pérez L., Gaucín Delgado J. M., Preciado-Rangel P., Gonzales Fuentes J. A., Ayala Garay A. V., Segura Castruita M. Á. (2018). The application of citric acid increases the quality and antioxidant capacity of lentil sprouts. Revista Mexicana de Ciencias Agrícolas, 9(Spe20), 4301–4309.

[bib106] Salihu R., Abd Razak S. I., Zawawi N. A., Kadir M. R. A., Ismail N. I., Jusoh N., Mohamad M. R., Nayan N. H. M. (2021). Citric acid: A green cross-linker of biomaterials for biomedical applications. European Polymer Journal, 146, 110271.

[bib107] Sanjay K., Sharma P. (1994). A highly performance fermentation process for production of citric acid from sugarcane molasses. Journal of Microbiology, 23, 211–217.

[bib108] Savić N., Schwank G. (2016). Advances in therapeutic CRISPR/Cas9 genome editing. Translational Research, 168, 15–21. 26470680 10.1016/j.trsl.2015.09.008

[bib109] Sawant O., Mahale S., Ramchandran V., Nagaraj G., Bankar A. (2018). Fungal citric acid production using waste materials: A mini-review. Journal of Microbiology, Biotechnology and Food Sciences, 8(2), 821–828.

[bib110] Shoukat A., Ahmad M., Akbar G. (1997). Citric acid production by *Aspergillus niger* by batch cultivation. Journal of Biotechnology, 7, 47–53.

[bib111] Show P. L., Oladele K. O., Siew Q. Y., Aziz Zakry F. A., Lan J. C.-W., Ling T. C. (2015). Overview of citric acid production from *Aspergillus niger*. Frontiers in Life Science, 8(3), 271–283.

[bib112] Shu P., Johnson M. J. (1948). The interdependence of medium constituents in citric acid production by submerged fermentation. Journal of Bacteriology, 56(5), 577–585. 16561608 10.1128/jb.56.5.577-585.1948 PMC518625

[bib113] Singh Dhillon G., Kaur Brar S., Verma M., Tyagi R. D. (2011). Recent advances in citric acid bio-production and recovery. Food and Bioprocess Technology, 4(4), 505–529.

[bib114] Sinko P. J. (2023). Martin's physical pharmacy and pharmaceutical sciences. Lippincott Williams & Wilkins.

[bib115] Soccol C., Vandenberghe L., Lebeault J. (1999). Production of citric acid by solid-state fermentation using *A. niger. Patent Pt. Br. DEINPI/PR*. 175.

[bib116] Soccol C. R., Vandenberghe L. P. (2003). Overview of applied solid-state fermentation in Brazil. Biochemical Engineering Journal, 13(2–3), 205–218.

[bib117] Soccol C. R., Vandenberghe L. P., Rodrigues C., Pandey A. (2006). New perspectives for citric acid production and application. Food Technology and Biotechnology, 44(2), 141–149.

[bib118] Suo Y., Li W., Wan L., Luo L., Liu S., Qin S., Wang J. (2023). Transcriptome analysis reveals reasons for the low tolerance of *Clostridium tyrobutyricum* to furan derivatives. Applied Microbiology and Biotechnology, 107(1), 327–339. 36418543 10.1007/s00253-022-12281-7

[bib119] Swain M. R., Ray R. C., Patra J. (2011). Citric acid: Microbial production and applications in food and pharmaceutical industries. In D. A. Vargas & J. V. Medina (Eds.), Citric acid: Synthesis, properties and applications(1st ed., pp. 97–118). Nova Science Publishers.

[bib120] Takatsuji W., Yoshida H. (1998). Adsorption of organic acids on weakly basic ion exchanger: Equilibria for binary systems. AIChE Journal, 44(5), 1216.

[bib121] Tomlinson N., Campbell J., Trussell P. C. (1950). The influence of zinc, iron, copper, and manganese on the production of citric acid by *Aspergillus niger*. Journal of Bacteriology, 59(2), 217–227. 15421950 10.1128/jb.59.2.217-227.1950 PMC385744

[bib122] Vandenberghe L. (2000). Development of process for citric acid production by solid-state fermentation using cassava agro-industrial residues. [PhD thesis/ Universite de Technologie de Compiegne, Compiegne, France].

[bib123] Vandenberghe L. P., Soccol C. R., Pandey A., Lebeault J.-M. (1999). Microbial production of citric acid. Brazilian Archives of Biology and Technology, 42(3), 263–276.

[bib124] Wennersten R. (1983). The extraction of citric acid from fermentation broth using a solution of a tertiary amine. Journal of Chemical Technology and Biotechnology. Biotechnology, 33(2), 85–94.

[bib125] Wu N., Zhang J., Chen Y., Xu Q., Song P., Li Y., Li K., Liu H. (2022). Recent advances in microbial production of L-malic acid. Applied microbiology and biotechnology, 106(24), 7973–7992. 36370160 10.1007/s00253-022-12260-y

[bib126] Wucherpfennig T., Hestler T., Krull R. (2011). Morphology engineering-osmolality and its effect on *Aspergillus niger* morphology and productivity. Microbial Cell Factories, 10(1), 1–15. 21801352 10.1186/1475-2859-10-58 PMC3178489

[bib127] Xie G., West T. (2009). Citric acid production by *Aspergillus niger* ATCC 9142 from a treated ethanol fermentation co-product using solid-state fermentation. Letters in Applied Microbiology, 48(5), 639–644. 19416466 10.1111/j.1472-765X.2009.02586.x

[bib128] Xu D.-B., Madrid C. P., Röhr M., Kubicek C. P. (1989). The influence of type and concentration of the carbon source on production of citric acid by *Aspergillus niger*. Applied Microbiology and Biotechnology, 30, 553–558.

[bib129] Xu Y., Zhou Y., Cao W., Liu H. (2020). Improved production of malic acid in *Aspergillus niger* by abolishing citric acid accumulation and enhancing glycolytic flux. ACS Synthetic Biology, 9(6), 1418–1425. 32379964 10.1021/acssynbio.0c00096

[bib130] Xue X., Bi F., Liu B., Li J., Zhang L., Zhang J., Gao Q., Wang D. (2021). Improving citric acid production of an industrial *Aspergillus niger* CGMCC 10142: Identification and overexpression of a high-affinity glucose transporter with different promoters. Microbial Cell Factories, 20(1), 1–13. 34446025 10.1186/s12934-021-01659-3 PMC8394697

[bib131] Yi M., Pen Q., Chen D., Pen L., Zhang M., Wen R., Mou X., Wang W. (1987). Extraction of citric acid by N,N-disubstituted alkyl amides from fermentation aqueous solution. Beiji Dax Xue, 4, 30–37.

[bib132] Yokoya F. (1992). Fermentação cítrica. (pp. 82–82).Série Fermentações Industriais.

[bib133] Yue J. J., Yuan J. L., Wu F. H., Yuan Y. H., Cheng Q. W., Hsu C. T., Lin C. S. (2021). Protoplasts: From isolation to CRISPR/Cas genome editing application. Frontiers in Genome Editing, 3, 717017. 34713263 10.3389/fgeed.2021.717017 PMC8525356

[bib134] Zhang W., Roy S., Assadpour E., Cong X., Jafari S. M. (2023). Cross-linked biopolymeric films by citric acid for food packaging and preservation. Advances in Colloid and Interface Science,314, 102886. 37002960 10.1016/j.cis.2023.102886

[bib135] Zheng Z., Coil A. L., Zehavi I. (2007). Galaxy evolution from halo occupation distribution modeling of DEEP2 and SDSS galaxy clustering. The Astrophysical Journal, 667(2), 760.

